# The impact of COVID‐19 on pregnancy and therapeutic drug development

**DOI:** 10.1111/bph.15582

**Published:** 2021-07-06

**Authors:** Allyah Abbas‐Hanif, Homira Rezai, Syed Faraz Ahmed, Asif Ahmed

**Affiliations:** ^1^ Division of Drug Development MirZyme Therapeutics Birmingham UK; ^2^ Department of Cardiology Chelsea and Westminster Hospital NHS Foundation Trust London UK; ^3^ School of Health Sciences University of Southampton Southampton UK

**Keywords:** cardiovascular diseases, clinical trials, COVID‐19, drug development, pre‐eclampsia, pregnancy, vaccines

## Abstract

Emerging data show that pregnant women with COVID‐19 are at significantly higher risk of severe outcomes compared with non‐pregnant women of similar age. This review discusses the invaluable insight revealed from vaccine clinical trials in women who were vaccinated and inadvertently became pregnant during the trial period. It further explores a number of clinical avenues in their management and proposes a drug development strategy in line with clinical trials for vaccines and drug treatments for the drug development community. Little is known of the long‐term effects of COVID‐19 on the mother and the baby. Our hypothesis that COVID‐19 predisposes pregnant women to pre‐eclampsia or hypertensive disorders during pregnancy is supported by a clinical study, and this may also adversely impact a woman's cardiovascular disease risk later in life. It may also increase a woman's risk of pre‐eclampsia in subsequent pregnancy. This is an ever‐evolving landscape, and early knowledge for healthcare providers and drug innovators is offered to ensure benefits outweigh the risks. COVID‐19 mRNA vaccines appear to generate robust humoral immunity in pregnant and lactating women. This novel approach to vaccination also offers new ways to therapeutically tackle disorders of many unmet medical needs.

**LINKED ARTICLES:**

This article is part of a themed issue on The second wave: are we any closer to efficacious pharmacotherapy for COVID 19? (BJP 75th Anniversary). To view the other articles in this section visit http://onlinelibrary.wiley.com/doi/10.1111/bph.v179.10/issuetoc

**Abbreviations**
COVID‐19: Coronavirus disease 2019
SARS‐CoV‐2: Severe acute respiratory syndrome coronavirus 2
CDC: Centers for Disease Control and Prevention
ACE‐2: angiotensin‐converting enzyme type‐2
Ang II: Angiotensin II
RAS: renin‐angiotensin system
TNF: Tumour necrosis factor
IL‐6: Interleukin‐6
IFN: interferon
CRP: C‐reactive protein
VEGF: Vascular Endothelial Growth Factor
sFlt‐1: Soluble Flt‐1
HO‐1: Heme Oxygenase‐1
CSE: Cystathionine γ‐lyase
H2S: hydrogen sulfide
miRNA: microRNA

## 1 INTRODUCTION

Coronavirus disease 2019 (COVID‐19) continues to drive an unprecedented global public health crisis. Multiple datasets have now demonstrated an increased risk of pregnant women with COVID‐19 having severe disease, requiring intensive care admission and increased maternal morbidity (Intensive Care National Audit & Research Centre [ICNARC], 2021; Patone et al., 2021; Vousden et al., 2021; Zambrano et al., 2020).

Pregnancy is a risky business. Pregnant women are at higher risk of morbidity and mortality compared with non‐pregnant women. The physiology of pregnancy presents well‐defined challenges to the maternal organism, and any stress or injury to the maternal or fetal system can put the life of the mother and the fetus in danger (Grimes, 1994). Pre‐eclampsia, one of the most common life‐threatening disorders among pregnant women, affects 5–8% of pregnancies (Duley, 2008). It is defined as the presence of new‐onset hypertension (BP ≥ 140/90 mmHg) without concurrent proteinuria, diagnosed after 20 weeks of gestation (Rana et al., 2019). Besides the direct life‐threatening complication in pregnancy, pre‐eclampsia can also increase the risk of developing cardiovascular complications (Behrens et al., 2017; Lykke et al., 2009), stroke (Bellamy et al., 2007) and vascular dementia (Basit et al., 2018) later in life.

The long‐term sequelae of COVID‐19 are evolving, and many organ systems may be involved. Protracted cardiovascular effects, especially in those with pre‐existing disease, may occur in COVID‐19 (Bandyopadhyay et al., 2020; Mitrani et al., 2020). Cardiovascular disease is the most common co‐morbidity associated with COVID‐19. The mortality rate in COVID‐19 patients with cardiovascular disease is higher compared with other co‐morbidities, such as diabetes, chronic kidney disease or cancer (Ssentongo et al., 2020). In this review, we hypothesise and provide a rationale as to why pregnant women with COVID‐19 may also experience a higher risk of cardiovascular disease, stroke and dementia in later life in a similar way to women with pre‐eclampsia (Ahmed et al., 2017). Indeed, a recent US study showed that pregnant women with COVID‐19 had higher incidence of myocardial infarction, venous thromboembolism and pre‐eclampsia events (Jering et al., 2021). These data support our hypothesis that pregnant women with moderate–severe COVID‐19 are predisposed to an increased long‐term cardiovascular risk later in life as well as increased risk of pre‐eclampsia in their subsequent pregnancy.

## 2 CLINICAL MANIFESTATIONS OF COVID‐19 IN PREGNANT WOMEN AND THEIR INFANTS

Women of reproductive age comprised 26% of all laboratory‐confirmed COVID‐19 cases in the United States between January and October 2021; 6.6% of whom were pregnant at the time of the test (Zambrano et al., 2020). The previous coronavirus and 2009 H1N1 pandemics (Rasmussen et al., 2012) resulted in high case fatality rates in pregnant women, leading to concern for women during pregancy with COVID‐19. The last Centers for Disease Control and Prevention (CDC) update, which included 409,462 women of reproductive age with symptoms and lab‐positive COVID‐19, reported not only an increased risk for ICU admission but also an increased maternal mortality rate with an adjusted risk ratio (aRR) of 1.7 (aRR = 1.7; 95% confidence interval [CI] = 1.2–2.4). This large, retrospective study does have limitations, with information on pregnancy status missing in 64.4% of cases, but it highlights a worrying change, which cannot be ignored, particularly in the light of more infectious strains of SARS‐CoV‐2. A recent, multicentre retrospective cohort study of 240 patients in the United States showed that the COVID‐19 case fatality rate in pregnant patients was 13.6‐fold higher compared with similarly aged‐matched individuals (Lokken et al., 2021). Recent data from the United Kingdom have shown that more pregnant women are being admitted to intensive care, especially in comparison with the first wave of COVID‐19 (ICNARC, 2021); 36 pregnant or recently pregnant women with COVID‐19 were reported to need critical care, between 1 November 2020 and 27 January 2021, 25 of whom were infected with the variant of concern B.1.1.7 (Patone et al., 2021). Pregnant patients with severe–critical COVID‐19 were at increased risk of perinatal complications (Metz et al., 2021).

The clinical presentation and risk factors for severe disease in pregnant women are similar to non gravid individuals. Risk factors like advanced age, obesity and pre‐existing cardiovascular disease, including hypertension and diabetes, play a significant role in increasing the chances of a more severe disease presentation and evolution (Allotey et al., 2020; Ellington et al., 2020). A recent retrospective study of 6380 pregnant women with COVID‐19 in the United States revealed that 212 (3.3%) needed intensive care and 9 died in hospital. COVID‐19 was associated with 21% higher rates of pre‐eclampsia (adjusted odds ratio [aOR], 1.21 [95% CI, 1.11–1.33]) and 17% more preterm birth (aOR, 1.17 [95% CI, 1.06–1.29]) (Jering et al., 2021).

Preterm delivery occurs in a higher proportion of pregnant women with COVID‐19 (Allotey et al., 2020; Mullins et al., 2021). The rationale to explain excess preterm and caesarean rates has been suggested to be related to the medical effects of COVID‐19 leading to obstetricians inducing or delivering before full term (Sentilhes et al., 2020). However, there may be additional biological plausibility. SARS‐CoV‐2 enters cells via the angiotensin‐converting enzyme type‐2 (ACE‐2), a cell‐surface protein, which is highly expressed during pregnancy (Bharadwaj et al., 2011; Li, Chen, et al., 2020; Pringle et al., 2011). The loss of the protective function of ACE‐2 results in downstream dysregulation and imbalance of angiotensin II (Ang II) and angiotensin 1–7 (Samavati & Uhal, 2020). Down‐regulation of ACE‐2 may lead to low vasodilatory levels of angiotensin 1–7 and the unopposed action of Ang II, potentially driving uterine contractions and subsequent preterm birth (Al‐Lami et al., 2021; Dhaundiyal et al., 2021). The unchecked effects of Ang II and subsequent up‐regulation of the renin‐angiotensin system (RAS)/Ang II pathway may also be involved in pre‐eclampsia and have an effect on fetal ACE‐2 reserves. Fortunately, the incidence of stillbirth and neonatal death does not seem to be higher than the background rate (Vousden et al., 2021) (Allotey et al., 2020; Zambrano et al., 2020).

In utero vertical transmission has been reported in case studies, and although rare, the SARS‐CoV‐2 genome has been found in umbilical cord blood, amniotic fluid, maternal vaginal mucosa and full‐term placenta (Fenizia et al., 2020). Fortunately, infection of neonates and infants is uncommon (Di Mascio et al., 2020). If neonates do become infected, most cases are asymptomatic or mild and outcomes are favourable (Chen et al., 2020; Zeng et al., 2020). Interestingly, both IgG and IgM antibodies against COVID‐19 have been found in seronegative neonates born to COVID‐19‐infected mothers (Carosso et al., 2020). As IgM antibodies cannot cross the placenta, the suggestion of a fetal immune response against the virus is possible (Carosso et al., 2020).

SARS‐CoV‐2 appears to attack the cardiovascular system, causing numerous cardiovascular complications. Over 20–30% of all adult patients hospitalised with COVID‐19 have some evidence of myocardial involvement (Bandyopadhyay et al., 2020; Mitrani et al., 2020; Puntmann et al., 2020). The virus induces an overactive inflammatory response with increased production of TNF, IL‐6 and IL‐1β leading to increased risk of vascular hyperpermeability (Jose & Manuel, 2020). Recent evidence also suggests that SARS‐CoV‐2 may also directly attack the vascular endothelium and disrupt the vascular barrier, leading to disseminated intravascular coagulation and inflammatory cell infiltration (Ackermann et al., 2020; Greene et al., 1981). Cardiac blood marker analysis from patients (*n* = 100) recovered from COVID‐19 infection showed ongoing myocardial inflammation in 60% of participants, independent of pre‐existing conditions, severity and overall course of the acute illness (Puntmann et al., 2020). Cardiac injury and inflammation are relatively commonly associated in patients hospitalised with COVID‐19, and such association is related to a higher risk of in‐hospital mortality. Although inflammation plays an important role in pathogenesis of COVID‐19, it is noteworthy that inflammation is also an important driver of cardiac pathological responses to stress, including heart failure where immune cells infiltrate the myocardium and release pro‐inflammatory cytokines. Studies have shown a positive correlation between high‐sensitive cardiac troponin T (hs‐cTnI) and biomarkers of inflammation and coagulation. Li, Jiang, et al. (2020) assessed 2068 patients with laboratory‐confirmed COVID‐19 and showed positive correlation of hs‐cTnI with IL‐6 and d‐dimer, which may suggest non‐specific cytokine‐mediated cardiotoxicity. In addition, troponin T levels were positively, linearly and significantly correlated with plasma high‐sensitivity C‐reactive protein (CRP) levels (Guo et al., 2020), indicating that myocardial injury may be associated with inflammatory pathogenesis during COVID‐19. Chronic myocardial inflammation may lead to long‐term consequences to cardiovascular system. Although myocarditis has been seen in previously healthy subjects including pregnant women with COVID‐19 (*n* = −2) (Juusela et al., 2020), this requires further investigation.

The hallmark of pre‐eclampsia is endothelial dysfunction due to defect in vascular protection (Ahmed et al., 2017). VEGF not only is an angiogenic factor (Ferrara et al., 1998) but also helps to maintain vascular homeostasis as it stimulates NO (Ahmed et al., 1997). The primary causative protein in the induction of pre‐eclampsia is the soluble form of VEGF receptor 1 (VEFGR‐1), known as sFlt‐1 (Ahmad & Ahmed, 2004; Levine et al., 2004; Maynard et al., 2003), which acts as an endogenous antagonist of VEGF. Although inflammation has been implicated as a cause of pre‐eclampsia (Redman et al., 1999), increasing evidence points to inflammation acting as an amplifier, rather than a causal factor (Ahmed et al., 2017; Ahmed & Ramma, 2015). Defects in two protective enzymes, haem oxygenase‐1 (Cudmore et al., 2007) and cystathionine γ‐lyase (Wang et al., 2013), lead to increases in sFlt‐1 in the mother's circulation and appear to cause pre‐eclampsia (Rezai et al., 2020; Saif et al., 2021).

Chronic hypertension is a major risk factor for pre‐eclampsia. Pre‐eclampsia is associated with chronic immune activation that leads to an increased production of inflammatory cytokines by pro‐inflammatory T cells (Bennett et al., 1998; Clark et al., 1998). The risk of developing chronic hypertension later in life increases by twofold to eightfold in women with hypertensive pregnancy disorder, compared with normotensive pregnancy (Behrens et al., 2017; Bokslag et al., 2017; Brouwers et al., 2018; Lykke et al., 2009). Women with a history of pre‐eclampsia have 3.7‐fold higher risk of developing hypertension 14 years after pregnancy, twice the risk of developing ischaemic heart failure after 11.7 years and twice the risk of getting a stroke 10.4 years after their pregnancy (Bellamy et al., 2007).

SARS‐CoV‐2 not only causes viral pneumonia but also has major implications for the cardiovascular system (Madjid et al., 2020). Accumulating evidence suggests that endothelial dysfunction and endothelial activation participate in COVID‐19 pathogenesis, which is also a hallmark of pre‐eclampsia and hypertension leading to further complications. Similar to pre‐eclampsia, COVID‐19 causes endothelial dysfunction by mediating leukocyte inflammation, inducing endothelial inflammation, altering vessel integrity and promoting a pro‐coagulative state (Bermejo‐Martin et al., 2020; Jin et al., 2020; Teuwen et al., 2020). COVID‐19 affects the lining of the blood vessels, and SARS‐CoV‐2‐mediated endothelial cell injury is an important effector of the virus, causing multi‐organ damage.

Patients with cardiovascular risk factors or with established cardiovascular and cerebrovascular disease are at increased risk of morbidity and mortality when suffering from COVID‐19 (Guzik et al., 2020). Moreover, COVID‐19 patients may develop cardiac injury or suffer from long‐term cardiovascular complications. We hypothesise that these damages may cause higher risk of cardiovascular disease, stroke and dementia in later life in a similar way to women with pre‐eclampsia. In addition, COVID‐19 may prove to be a risk factor for pregnancy complications such as pre‐eclampsia. Indeed, a retrospective cohort study of almost 2000 births in which almost 100 women were COVID‐19 positive found a twofold higher risk of hypertensive disorders of pregnancy (hazard ratio [HR], 1.93; 95% CI, 1.13–3.31) (Rosenbloom et al., 2021). Although this is a limited single‐institution study, it confirms our hypothesis that women who are exposed to SARS‐CoV‐2 infection may be at a higher risk of developing pre‐eclampsia during pregnancy. Pre‐eclampsia is on the rise and remains an unmet medical need that urgently requires preventative therapeutics. Although there are no treatments available to prevent or treat pre‐eclampsia in the market, a novel hydrogen sulfide‐releasing molecule has shown to prevent pre‐eclampsia in animal models (Rezai et al., 2020; Saif et al., 2021; Sanchez‐Aranguren et al., 2020).

## 3 RECOMMENDATIONS TO ALLOW INCLUSION OF PREGNANT WOMEN IN DRUG DEVELOPMENT PLANS DURING A PANDEMIC

**FIGURE 1:**
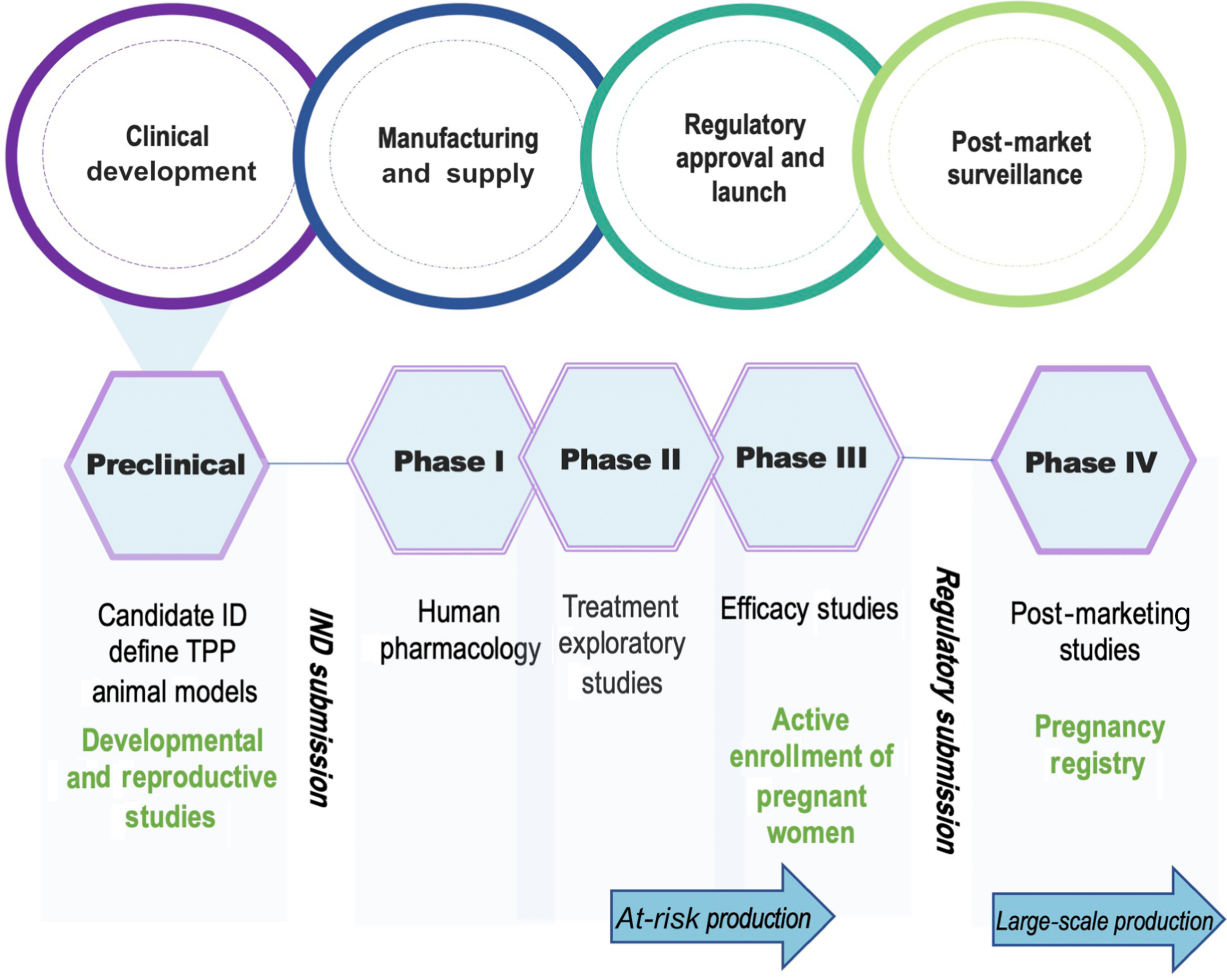
Diagram to illustrate the recommendations for inclusion of pregnant women in vaccine and drug development during the COVID‐19 pandemic. Vaccine and drug development during the COVID‐19 pandemic has accelerated, with phases overlapping and at‐risk vaccine production during clinical trials. Developmental and reproductive studies should take place as early as possible in the clinical development programme, ideally during preclinical evaluation of diverse animal models. Target product profile (TPP) for the product lead (candidate ID) should be discussed and developed with regulatory bodies during preclinical phase. Inclusion of pregnant women in Phase III with rolling review by data monitoring and safety committees, as well as post‐marketing studies, will provide clinicians with the data needed to make evidence‐based decisions

**FIGURE 2:**
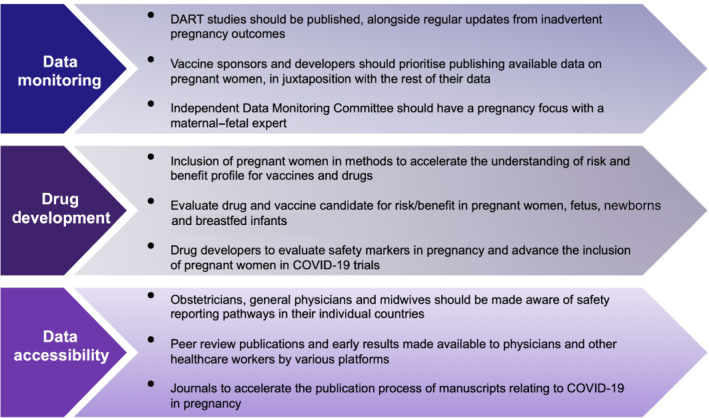
Recommendations for vaccine and drug development and accessibility for pregnant patients with COVID‐19. Data on COVID‐19 vaccine and treatment should be monitored, and the developmental and reproductive toxicity (DART) data should be made available

In this global emergency, methods to accelerate the understanding of risk/benefit profiles must ensure early consideration of the inclusion of special groups, such as pregnant women, in clinical development plans. Pregnant women should be afforded the option to participate in clinical trials, just like any other eligible subjects, and this is supported by regulators (European Medicines Evaluation Agency [EMEA], 1996; Food and Drug Administration [FDA], 2006; International Conference on Harmonisation [ICH], 2009) and the World Health Organization (WHO) (Taylor et al., 2021). Key strategies to allow safe participation of pregnant women into an investigational programme are discussed in Figures 1 and 2 and include the conduct of developmental and reproductive toxicity (DART) studies, fully utilising already established pregnancy and postmarketing registries (ICH, 2009) and releasing post approval data in a timely manner.

## 4 VACCINES

The effectiveness of any SARS‐CoV‐2 immunisation during pregnancy depends upon the ability and efficacy of the vaccine to induce the body's protective immune responses, coupled with the timing of vaccine delivery during the gestational period. Drug developers seek to harmonise peak vaccine response with the time of greatest vulnerability in pregnancy. This needs to be offset by any consequences for fetal programming and adverse outcomes. Phase II and III clinical trials have strict processes and checkpoints to ensure patient safety. These include investigators trained on the study protocol and safety reporting, internal development and safety physicians who oversee process and review (usually) blinded data in a real‐time manner. Independent Data Monitoring Committees (DMCs) are tasked with cumulative review of unblinded data, and regulatory compliant pharmacovigilance and safety reporting pathways are well established globally (EMEA, 1996; FDA, 2006). During the pandemic, regulators have undertaken rolling reviews (European Medicines Agency [EMA], 2021) of vaccine data and independent vaccine committees offer input. These platforms could be modified to have a pregnancy focus with additional maternal–fetal experts on the DMC.

There are over 200 vaccines in development, with 82 vaccines in clinical stage evaluation (WHO, 2021). COVID‐19 vaccines are now being deployed globally and include the Pfizer‐BioNTech and Moderna vaccines, which use the instructions from mRNA. In contrast, vaccines from Janssen and AstraZeneca‐University of Oxford's viral vector, and Sinovac (CoronaVac) and Sinopharm (BBIBP‐CorV) both use inactivated virus (Table 1). None of the vaccine premarketing studies recruited pregnant women (although Janssen do enrol breastfeeding women), leading to limitations in safety data in a group that is potentially at risk. The mRNA and DNA vaccine delivery system, using a replication‐deficient adenoviral vector or lipid‐based, biodegradable carriers (Park et al., 2021), has opened the potential for the development of new therapeutic agents using mRNA, DNA and microRNA (miRNA). None of the compounds are live‐attenuated vaccines and so could be used in pregnancy. No data are available on the use of COVID‐19 mRNA vaccines in pregnant women, but Zika animal studies demonstrated that the mRNA vaccine was safe in pregnant mice and prevented vertical transmission of Zika virus (Richner et al., 2017).

**TABLE 1:** Vaccines and their pregnancy‐specific postmarketing studies

Developer	Vaccine name and component	Current stage	Eligibility criteria for pregnant women Phase 1–3	Pregnancy‐specific postmarketing studies
Pfizer‐BioNTech	BNT162b2 (mRNA of full‐length spike protein in a lipid nanoparticle)	Postmarketing	Pregnant/breastfeeding excluded	C4591001^a^: a Phase 1/2/3, placebo‐controlled, randomised, observer‐blind, dose‐finding study to evaluate the safety, tolerability, immunogenicity and efficacy of SARS‐CoV‐2 RNA vaccine candidates against coronavirus disease 2019 (COVID‐19) in in healthy individuals C4591015: a Phase 2/3, placebo‐controlled, randomised, observer‐blind study to evaluate the safety, tolerability and immunogenicity of a SARS‐CoV‐2 RNA vaccine candidate (BNT162b2) against COVID‐19 in healthy pregnant women 18 years of age and older C4591008: post‐emergency use authorisation observational cohort study to evaluate the safety of SARS‐CoV‐2 RNA vaccine in healthcare workers: a primary data collection active surveillance study C4591011: safety surveillance of the Pfizer COVID‐19 vaccine in the US Department of Defence population following emergency use authorisation C4591012: post‐emergency use authorisation active surveillance of adverse events of special interest among individuals in the Veterans Affairs health system receiving Pfizer‐BioNTech COVID‐19 vaccine
AstraZeneca‐University of Oxford	AZD1222 ChAdOx1 nCoV‐19 (replication‐deficient adenovirus type 5 vector‐expressing full‐length spike protein)	Postmarketing	Pregnant/breastfeeding excluded	D8111R00003, D8111R00004^a^: enhanced active surveillance A Phase IV enhanced active surveillance study of people vaccinated with AZD1222 No study code: AZD1222 pregnancy registry of women exposed to AZD1222 immediately before or during pregnancy
Moderna	mRNA‐1273 (mRNA of full‐length spike protein in a lipid nanoparticle)	Postmarketing	Pregnant/breastfeeding excluded	NCT04470427^a^: sponsor has committed to a 3‐year passive pregnancy registry. A US‐based prospective observational study may take place
Johnson & Johnson/Janssen Pharmaceuticals	Ad26.COV2.S or JNJ‐78436735 (replication‐deficient adenovirus type 26 vector‐expressing full‐length spike protein)	Phase 3 trial in progress, interim results expected Q1 2021	Lactating women are permitted in Phase 3 study Pregnant women excluded but Ad26+ has had exposure in Ebola (1000 patients)	Details awaited
Novavax	NVX‐CoV2373 (a ‘nanoparticle’ of trimeric full‐length recombinant spike protein formulated in Matrix‐M1 adjuvant)	Phase 3 trial ongoing, interim data expected Q1 2021	Pregnant/breastfeeding excluded	Details awaited
Sinovac	CoronaVac (vaccine with inactivated virus)	Approved in China. Emergency use in other countries	Pregnant/breastfeeding excluded	Details awaited
Sinopharm	BBIBP‐CorV (vaccine with inactivated virus)	Approved in China. Limited use in UAE	Pregnant/breastfeeding excluded	Details awaited

*Note*: Vaccine developers have shown commitment to conduct postmarketing safety and active surveillance studies and registries in pregnant women. Pfizer‐BioNTech, AstraZeneca‐University of Oxford and Moderna have published their proposed specific postmarketing studies, whereas other developers such as Johnson & Johnson/Janssen Pharmaceuticals and Novavax are in late‐stage clinical trials.

^a^ Ongoing Phase 4 studies of original Phase1/2/3 subjects. Surveillance is planned on average for 2 years following Dose 2. The sponsors will release details of all details of all inadvertent pregnancies, which occurred prior in premarketing clinical studies on a periodic basis to regulators.

Inactivated virus technology is a ‘tried and tested’ methodology for developing vaccines and historically has a good safety profile in both the short‐term and long‐term evaluation. This approach may be safer for pregnant women and for future generations of COVID‐19 vaccines, as the virus mutates. The use of ‘inactivated whole virus’ used by Sinovac from Sinopharm may offer greater protection than vaccines that only target ‘a single spike protein’. The Sinopharm vaccine has gone into the arms of over 12 million people around the world including multi‐ethnic groups in the United Arab Emirates, but the company has yet to publish their Phase III clinical data.

Inclusion of pregnant women in vaccine trials, especially those looking to rapidly deliver results, is challenging but maternal immunisation, such as influenza vaccination, is a highly successful tool, which is critically a dual protection to mother and infant (Shakib et al., 2016). Transplacental transfer of SARS‐CoV‐2 IgG antibodies from seropositive pregnant women after natural infection is recognised (Flannery et al., 2021). Cases of passive immunity through IgG transfer from vaccinated pregnant mothers have now also been reported and provide an encouraging additional benefit for COVID‐19 vaccines (Gilbert & Rudnick, 2021; Gray et al., 2021), especially as the emergence of new viral variants of concern leads to growing perturbations of vaccination programmes (Flannery et al., 2021).

Pre‐approval vaccine studies have included thousands of female subjects. Extrapolating data from women of childbearing age in clinical trials and those who inadvertently become pregnant offers invaluable insights. Women accounted for approximately 49.4% of Pfizer‐BioNTech BNT162b2 Phase III trial participants and, as of 14 November 2020 data cut, 23 participants reported intercurrent pregnancy (12 subjects in the vaccine group) (FDA, 2020a). The Moderna mRNA‐1273 trial reported 13 reports of inadvertent pregnancies (six cases in the vaccine group as of 2 December 2020) (FDA, 2020b). The Oxford‐AstraZeneca AZD1222 Phase III trial has reported 21 pregnancies (12 in the vaccine group as of 21 November 2020). Of these pregnancies, five ended in spontaneous abortion, two in the AZD1222 group (Medicines & Healthcare Products Regulatory Agency [MHRA], 2020). Outcomes of the total of 57 inadvertent pregnancies cases are actively being followed. A recently reported study in which 84 pregnant, 31 lactating and 16 non‐pregnant women of reproductive age were the recipients of the COVID‐19 mRNA vaccination, showed that vaccine‐induced antibody titres were equivalent in pregnant and lactating subjects, compared with non‐pregnant women. Clearly, COVID‐19 mRNA vaccines generate robust humoral immunity in pregnant and lactating women. The authors concluded that vaccine‐induced immune responses were significantly greater than the response to natural infection and immune transfer to neonates occurred via placenta and breastmilk (Gray, Bordt, et al., 2021).

Limited details about the 57 inadvertent pregnancies in the clinical trials are currently reported, and definitive strategies to help provide data such as large‐scale postmarketing pregnancy studies are important but take time to deliver data. Other options to help provide data in a timely manner include conducting DART studies early in a clinical development plan, particularly during the preclinical phase, and are recommended by regulators (ICH, 2009). Many structural and functional parallels exist between human and animal models, providing a valuable platform for evaluating safety and efficacy of potential drug candidates. Rodents, for example, have a haemochorial placenta, short gestation and large litters, making them ideal for performing high‐throughput screening of candidate therapeutic agents (Grigsby, 2016). Animal models are also important for identifying drug‐related teratogenic effects and their timing during pregnancy. For example, rodent and rabbit models were instrumental in demonstrating that the drug‐related teratogenic effects of artemisinin‐based combination therapies for malaria were limited to the first trimester (Clark, 2009). Animal studies can provide the first insights into the optimum window to administer COVID‐19 vaccines and if any teratogenic effects exist. DART studies with BNT162b2, Moderna and limited data from AstraZeneca have revealed no vaccine‐related effects on female fertility, pregnancy or embryo–fetal development (FDA, 2020a). Data from DART studies can be extrapolated to other compounds with similar mechanisms of action. Interim data from Janssen's Ad26.COV2.S are now available (Sadoff et al., 2021). This programme offers a unique opportunity for pregnant women, as it is a single‐dose strategy and Ad26+ has been trialled in pregnant women with exposure to Ebola (1000 patients). Pregnant women are excluded from the Phase III COVID‐19 trial, but breastfeeding women are eligible. Janssen alongside other sponsors should prioritise publishing available data on pregnant women, in juxtaposition, with the rest of their data. The early completion of DART studies is critical and could offer a catalytic step to earlier recruitment of pregnant women in the development plans for vaccines and novel agents.

The data from currently approved vaccines have not indicated any safety concerns, allowing the regulatory bodies like the MHRA and FDA to recommend that clinicians undertake case‐by‐case assessments for the use of COVID‐19 vaccines by pregnant women, particularly those with high‐risk co‐morbidities (Royal College of Obstetricians and Gynaecologists [RCOG], 2020a). Pfizer‐BioNTech has commenced its post‐approval study to evaluate the safety, tolerability and immunogenicity of BNT162b2 in preventing COVID‐19 in 4000 healthy pregnant women (ClinicalTrials.gov, 2021a). The programme uses an overlapping Phase II–III study design, as outlined in Figure 1. Conventionally, these data would not be available for many months; we encourage the sponsor to work with competent authorities to release rolling data in a timely manner. The trial will enrol women between 24 and 34 weeks of gestation and answer questions on timing of vaccine delivery during the gestational period. The trial and post‐marketing pregnancy registries are essential to answer clinical questions in relation to maternal hyperthermia which, particularly in the first trimester, is associated with neural tube defects and other congenital abnormalities (Graham, 2020). All three licensed vaccines have reported pyrexia, a symptom of vaccine reactogenicity, as very common (>10%). Clinicians currently need to infer conclusions, such as potentially avoiding immunisation in the first trimester, until further data are available. Other open clinical questions include how long immune protection lasts and if a vaccine is given before conception, will it safeguard the whole pregnancy. To aid decision making, DART studies should be published, alongside regular updates from inadvertent pregnancy outcomes and a rolling review of all post‐marketing studies. Regulators have asked that sponsors commit to post‐marketing safety and active surveillance studies and registries in pregnant women. Currently ongoing and prospective studies are listed in Table 1.

In the United States, vaccines are currently being prioritised in a phased approach, with healthcare workers participating in Wave 1a. Approximately 75% of the US healthcare workers are women, and it is estimated that 330,000 women could be pregnant or at post‐partum during the first phase of vaccine implementation. This and other large‐scale data from pregnancy‐specific cohorts will further the understanding of whether pregnancy alters the effectiveness of treatments and if there are any effects on fetal development and breastfeeding. These women will all be followed up via pregnancy registries or post‐marketing studies. Rare adverse drug reactions will, however, only be picked up once the vaccines enter general use, which means that pregnancy registries, specific to vaccines, are essential. Obstetricians, general physicians and midwives should be made aware of safety reporting pathways in their individual countries to facilitate this process.

SARS‐CoV‐2 variants have arisen in several locations including the United Kingdom, South Africa, Brazil and the United States. Vaccines developed against the original virus have been found to be less effective against B.1.351, first detected in South Africa (CDC, 2021; Mahase, 2021). Several companies are updating their vaccines to target new variants. Future mutations may further increase transmission and also, worryingly for pregnant women, virulence. Drug developers from the 250 other vaccines in development should aim to evaluate safety markers in pregnancy and follow the successful precedent set by the approved vaccines to advance the inclusion of pregnant women in COVID‐19 trials.

## 5 MEDICAL TREATMENTS

Vaccination roll‐outs offer an accepted strategy out of the COVID‐19 pandemic, but active therapy to tackle the severe disease that pregnant women risk, is urgently needed. Strategies to repurpose drugs with known safety profiles in pregnancy offer a useful first step but need to go further. Figure 2 summarises our recommendations for vaccine and drug development and accessibility for pregnant patients with COVID‐19. The RECOVERY Trial is an open‐label, platform study that is currently enrolling pregnant women into its synthetic neutralising antibodies (Regeneron's REGN10933 and REGN10987) arms (RECOVERY Trial, 2020). The study design includes protocol‐specific pregnancy documents prepared by a panel of maternal–fetal experts and a pregnancy lead appointed to work with the principal investigator at each site. So far, the study has shown a mortality benefit of low‐dose dexamethasone in patients with COVID‐19 who required respiratory support, which is now a cornerstone for COVID‐19 management (Horby et al., 2020). No pregnancy‐associated adverse outcomes have been reported. RECOVERY also demonstrated a survival benefit for hypoxic hospitalised COVID‐19 patients with systemic inflammation (CRP at or above 75 mg·L^−1^). The data from pregnant women in this study arm of the trial appear to be limited, but as there is no evidence of teratogenicity, tocilizumab should be considered in pregnant women with both hypoxia and systemic inflammation (Horby et al., 2021a). Several trials and observational studies looking at convalescent plasma have included pregnant women (Joyner et al., 2021). Results have been conflicting, but the RECOVERY Trial ended early in mid‐January 2021 (Horby et al., 2021b), following no improvement in survival in 1398 patients randomised to convalescent plasma.

Remdesivir is an antiviral drug, originally developed to treat Ebola and Marburg virus infections, and included use in pregnant women (Mulangu et al., 2019). Its use has demonstrated a reduced time to recovery in COVID‐19, particularly in those requiring supplemental oxygen (Beigel et al., 2020). The ACTT‐1 trial did not include pregnant women, but there were no significant safety concerns reported in women of childbearing potential (Beigel et al., 2020). Its subsequent use by hospitalised pregnant women suffering with severe COVID‐19 disease has occurred via expanded access programmes in five countries with favourable outcomes (McCoy et al., 2020). The US National Institutes of Health has recently commenced a Phase I pharmacokinetic study to understand the effects of remdesivir in pregnancy (ClinicalTrials.gov, 2021b). The use of steroids and remdesivir has not led to label expansion, but both are now included in national guidelines (RCOG, 2020b).

There are several trials looking at the use of IFN‐α and IFN‐β in COVID‐19, mostly in addition to antivirals. Results with injectable forms have been disappointing (Monk et al., 2020). An investigational inhaled nebulised IFN‐β1a (SNG001) has however shown some promise. When administered to hospitalised patients with COVID‐19 in a Phase IIb study, the likelihood of recovery by Day 15, compared with placebo, was increased (Monk et al., 2020). Several studies (mostly in multiple sclerosis) have shown no increase in congenital abnormalities with IFN use (Hellwig et al., 2020); this coupled with the potential to bypass the placenta makes nebulised IFN‐β1a an attractive option for pregnant women. DART studies should also be conducted with this investigational product to allow further insights to the risk/benefit in pregnant women. The use of other agents such as monoclonal antibody therapies has shown some promise in clinical trials, and case reports of use in pregnant women are growing (Gordon et al., 2021; Naqvi et al., 2020). With the higher risk of severe COVID‐19 in pregnant women, in the absence of absolute contraindications, it is reasonable to consider pregnant women for inclusion in clinical trials of these therapeutic approaches.

The COVID‐19 pandemic has fuelled innovations never previously seen. The collaborative links between industry, academic, regulatory and government bodies have allowed clinical development programmes, which usually take a decade, to be delivered in less than a year. This momentum and lessons learned for efficient and patient‐centred drug development plans should now go beyond SARS‐CoV‐2 and to other infectious states, which severely affect pregnancy and neonates such as Zika and respiratory syncytial virus.

## 6 CONCLUSION

Pregnant women experience more severe COVID‐19, compared with non‐pregnant women. Immunisation and treatment strategies for pregnant women during the COVID‐19 pandemic should be tailored to optimise protection for both mother and infant. The race to find appropriate treatments and vaccines for COVID‐19 is progressing swiftly but, with the urgency of more transmissible variants and more intensive care admissions, strategies to enrol pregnant women earlier into clinical development plans should be utilised. Figure 2 summarises our recommendations for vaccine and drug development and accessibility for pregnant patients with COVID‐19. This, coupled with industry‐supported pregnancy registries and close collaboration with regulators and government bodies, will allow pregnant women to have access to investigational clinical trials, while mitigating potential risks.

SARS‐CoV‐2 mediates leukocyte inflammation, induces endothelial inflammation, alter vessel integrity and promote pro‐coagulative states. Experiencing COVID‐19 may increase the risk of developing pre‐eclampsia during pregnancy. Due to the similarities in the pathogenesis of COVID‐19 and pre‐eclampsia, we suggest that COVID‐19 may increase the risk of pregnant women experiencing further health complications such as cardiovascular disease, stroke and dementia later in life. COVID‐19 has been shown to cause adverse outcomes of pregnancy. Greater morbidity and mortality during pregnancy are associated with symptomatic COVID‐19. Severe and critical COVID‐19 can lead to adverse pregnancy outcome such as preterm birth. The primary hallmark of pre‐eclampsia is endothelial dysfunction due to defects in vascular protection. Pre‐eclampsia is an independent risk factor for maternal cardiovascular disease, a disorder in which endothelial dysfunction is also involved. Likewise, COVID‐19 may prove to be an independent risk factor for pre‐eclampsia and cardiovascular complications. This will need scientific and clinical investigation to prove or disprove our proposed hypothesis.

### 6.1 Nomenclature of targets and ligands

Key protein targets and ligands in this article are hyperlinked to corresponding entries in http://www.guidetopharmacology.org and are permanently archived in the Concise Guide to PHARMACOLOGY 2019/20 (Alexander, et al., 2019; Alexander, Fabbro et al., 2019a, b).

## AUTHOR CONTRIBUTIONS

All authors contributed to writing and editing this manuscript. AAH and HR drafted the preliminary manuscript and AA proposed the hypothesis that COVID‐19 will contribute to increasing the incidence of pre‐eclampsia and pregnancy complications worldwide.

## CONFLICT OF INTEREST

The authors declare no conflicts of interest.
